# The helminth derived peptide FhHDM-1 redirects macrophage metabolism towards glutaminolysis to regulate the pro-inflammatory response

**DOI:** 10.3389/fimmu.2023.1018076

**Published:** 2023-01-25

**Authors:** Susel Loli Quinteros, Eliana von Krusenstiern, Nathaniel W. Snyder, Akane Tanaka, Bronwyn O’Brien, Sheila Donnelly

**Affiliations:** ^1^ School of Life Sciences, Faculty of Science, University of Technology Sydney, Ultimo, NSW, Australia; ^2^ Lewis Katz School of Medicine, Temple University, Philadelphia, PA, United States

**Keywords:** *Fasciola hepatica*, helminth defence molecule, macrophage, immunometabolism, glutaminolysis, immune regulation, alpha-ketoglutarate (α-KG), fatty acid oxidation (FAO)

## Abstract

We have previously identified an immune modulating peptide, termed FhHDM-1, within the secretions of the liver fluke, *Fasciola hepatica*, which is sufficiently potent to prevent the progression of type 1 diabetes and multiple sclerosis in murine models of disease. Here, we have determined that the FhHDM-1 peptide regulates inflammation by reprogramming macrophage metabolism. Specifically, FhHDM-1 switched macrophage metabolism to a dependence on oxidative phosphorylation fuelled by fatty acids and supported by the induction of glutaminolysis. The catabolism of glutamine also resulted in an accumulation of alpha ketoglutarate (α-KG). These changes in metabolic activity were associated with a concomitant reduction in glycolytic flux, and the subsequent decrease in TNF and IL-6 production at the protein level. Interestingly, FhHDM-1 treated macrophages did not express the characteristic genes of an M2 phenotype, thereby indicating the specific regulation of inflammation, as opposed to the induction of an anti-inflammatory phenotype *per se*. Use of an inactive derivative of FhHDM-1, which did not modulate macrophage responses, revealed that the regulation of immune responses was dependent on the ability of FhHDM-1 to modulate lysosomal pH. These results identify a novel functional association between the lysosome and mitochondrial metabolism in macrophages, and further highlight the significant therapeutic potential of FhHDM-1 to prevent inflammation.

## Introduction

The proliferation, differentiation, and function of immune cells can be directly or indirectly modulated by reprogramming the intrinsic metabolic pathways in immune cells. Thus, the concept of immunometabolism has proposed that reprogramming the metabolic activity of macrophages offers a mechanism to selectively and specifically, regulate inflammation and immunity (1–4). In their resting state, macrophages generally exhibit low biosynthetic demands predominantly relying on highly efficient metabolic pathways, such as mitochondrial oxidative phosphorylation (OXPHOS). In response to stimulation with bacterial ligands, such as LPS, the metabolic activity of macrophages switches from OXPHOS to aerobic glycolysis, which facilitates the fast ATP production required to support the pro-inflammatory, antimicrobial activity of M1 macrophages (5, 6). Subsequently, during the resolution of inflammatory responses or the onset of immunosuppression, there is a reduction in glycolytic flux and a metabolic shift back to OXPHOS (7, 8).

The induction of glycolytic activity in macrophages is not limited to microbial ligands. Increased glycolytic flux, and functional activation of an M1 phenotype, can also be induced by metabolites, oxidized low-density lipoprotein, glucose, and signals released after cellular damage (9–11). In these scenarios, and in contrast to the beneficial effect of M1 macrophage induction during microbial infection, the activation of glycolytic pathways by endogenous or exogenous sterile stimuli leads to an enhanced pro-inflammatory response, which mediates the deleterious effects associated with immune-mediated disease (12–14). Thus, the selective modulation of metabolic pathways offers a new therapeutic strategy to skew macrophages towards a less inflammatory phenotype that would prevent tissue damage and the initiation of the inflammatory responses responsible for the progression of inflammatory/autoimmune disease.

Compelling epidemiological evidence demonstrates a robust inverse correlation between the prevalence of endemic helminth infections and the incidence of immune-mediated disease globally (15, 16). Multiple animal-based experimental studies and human investigations have corroborated the potent ability of helminth infection, and/or treatment with their excretory-secretory products, to skew immune responses towards an anti-inflammatory/tolerogenic profile to prevent/reverse inflammatory disease (17, 18). Further, it has been shown that this protective effect can be simulated by the adoptive transfer of macrophages that have been exposed to helminth products and induced to undergo a functional modification (19–22). Accordingly, understanding the mechanisms by which helminth molecules modulate the functional phenotypes of macrophages opens new avenues to prevent and treat human immune-mediated disease.

Helminth infection is typically characterised by polarisation towards a Th2 type immune response in which populations of anti-inflammatory (alternatively activated) M2 macrophages predominate and the differentiation of pro-inflammatory M1 is inhibited. These macrophages display increased mitochondrial OXPHOS metabolism. Maintenance of this metabolic pathway and the M2 phenotype was initially considered to be dependent on fatty acid oxidation (FAO), fuelled by lysosomal lipolysis of fatty acids (23). However, it is now evident that while the IL-4 induced expression of M2 genes simply requires a threshold of ATP, which can be provided by OXPHOS or glycolysis (24), the anti-inflammatory functional activity of macrophages is mediated only by OXPHOS, which can be fuelled by Acetyl-CoA or glutamine (25–27). The administration of IL-4 to mice fails to fully replicate the metabolic reprogramming of macrophages observed during helminth infection, suggesting that additional host or parasite derived factors also have the capacity to reprogram the metabolic signature of macrophages to induce a functional phenotype beneficial to the prevention of inflammation (28). In addition, it is possible that helminth induced reprogramming of macrophage metabolic activity mediates the observed improvement in overall whole-body metabolism seen during chronic infection of humans (29), and experimental work in mouse models of metabolic disease (30–33). Thus, exploiting the mechanisms employed by parasites to reprogram macrophage metabolism, represents an effective strategy for treatment of a range of immune-mediated disease and metabolic disorders.

We have previously identified the helminth defence molecule secreted by the liver fluke *Fasciola hepatica* (FhHDM-1) as a peptide with therapeutic efficacy in murine models of type 1 diabetes and multiple sclerosis (34, 35). Interestingly, after injection FhHDM-1 preferentially interacted with macrophages *in vivo* and reduced their capacity to secrete pro-inflammatory cytokines in response to stimulation (34). This observation coupled with the central and early role of pro-inflammatory macrophages in both diseases, suggested that the protective effect of FhHDM-1 was mediated by the modulation of macrophage phenotypes. Subsequent *in vitro* analyses revealed that after interacting with the macrophage membrane, FhHDM-1 was actively internalized and localized to endolysosomes where it inhibited vacuolar ATPase (vATPase), an enzyme central to the regulation of lysosomal pH (36). Consequently, pro-inflammatory activities that are dependent upon an acidic lysosomal pH, such as NOD-like-receptor (NLR)-P3 activation, were inhibited (37). Given that the activation of NLRP3 is tightly regulated by cellular metabolism (38), we hypothesised that changes in the utilisation of specific metabolic pathways within macrophages may underpin the biological activity of FhHDM-1. This presents a significant therapeutic opportunity to specifically, and selectively, regulate the inflammatory behaviour of macrophages.

## Materials and methods

### Production of parasite-derived peptides by chemical synthesis

Synthetic peptides corresponding to the sequence of the mature full-length native FhHDM-1 (39) and an inactive mutant derivative [NHP (36);], were synthesised to 95% purity and verified to be endotoxin free (GLBiochem, Shanghai, China). The peptides were solubilised in sterile, endotoxin-free water (Baxter) aliquoted, and stored at -80°C until use.

### Isolation and culture of bone marrow-derived macrophages

Bone marrow was harvested from 6-week-old C57BL/6 mice and cultured at 37°C/5% CO_2_ in RPMI 1640 Medium (Gibco) containing 10% v/v heat-inactivated FCS (Gibco), 2-mercaptoethanol (Sigma-Aldrich) and 5% penicillin/streptavidin (Life Technology). The cells were supplemented with 50ng/ml recombinant macrophage colony stimulating factor (Miltenyi Biotec) on days 1 and 3 of culture to stimulate the differentiation of monocytes to macrophages. At day 6, differentiated BMDMs were harvested and used for experiments. Culture and stimulation conditions for each experiment are described in the respective figure legends. Ethical approval for these studies was granted by the University of Technology Sydney (UTS) Animal Care and Ethics Committee (Approval Number: ETH18-2257) and experiments were conducted in accordance with the approved guidelines to be compliant with The Australian Code for the Care and Use of Animals for Scientific Purposes.

### Isolation of murine peritoneal macrophages

C57BL/6 mice (6 weeks old) were administered either 10μg of FhHDM-1 in 100μl sterile saline, or saline, by an intraperitoneal injection. One hour post injection, peritoneal cells were harvested by lavage with 5ml sterile saline, and peritoneal macrophages were isolated by negative selection (Miltenyi Biotec). The purified macrophages were seeded overnight at 1x10^5^ cells per well before analysis of metabolic activity. Ethical approval for these studies was granted by the University of Technology Sydney (UTS) Animal Care and Ethics Committee (Approval Number: ETH21-5823) and experiments were conducted in accordance with the approved guidelines to be compliant with The Australian Code for the Care and Use of Animals for Scientific Purposes.

### Quantification of cytokine secretion by macrophages

The levels of cytokines secreted by BMDMs in response to stimulation with LPS (20ng/mL) were quantified using ELISA kits (BD Bioscience), according to the manufacturer’s instructions. Absorbance at 450nm was quantified using a Tecan plate reader. Absorbance readings were corrected for background absorbance, and then used to calculate cytokine concentrations by extrapolation from a standard curve.

### RNA extraction, cDNA synthesis and qPCR

Total RNA was isolated from macrophages using the Isolate II RNA mini kit (Bioline/Life Science), according to the manufacturer’s instructions. cDNA was reverse transcribed using a mixture of random hexamer (Life Technologies) and 10mM dNTP (Life Technologies). After 5 min in an Eppendorf Mastercycler at 65°C, 10 µL of reverse transcriptase master mixture (10X RT buffer, 25mM MgCl_2_, 0.1M DTT, RNAse OUT™ and SuperScript III Reverse Transcriptase - Superscript III First Strand Synthesis Kit [Thermofisher Scientific]) was added to samples. qRT-PCR analysis was performed using TaqMan gene expression master mixture and TaqMan primers (Applied Biosystems; **Supplementary Table S1**). The reaction was run on a Quant Studio^™^ 12K Flex machine for 40 cycles.

The software programmes GeNorm (40), NormFinder (41) and Best-Keeper (42) were used to identify the optimal housekeeping gene to calculate the relative expression levels of genes by qRT-PCR. For this, the raw Ct values were transformed to different input formats for GeNorm and NormFinder analyses. For analysis using BestKeeper software, raw Ct values were used. Based on this analysis, differential gene expression was calculated after normalization to the Stx5a (Applied Biosystems) housekeeping gene.

### Analyses of metabolic pathways by measuring extracellular flux

The Seahorse XF24 Extracellular Flux Analyzer (Agilent Technologies) was used to measure the extracellular acidification rate (ECAR) and oxygen consumption rate (OCR). Following treatment with LPS, IL-4, or HDM peptides (as described in the figure legends) BMDMs (1x10^5^ cells per well) were rinsed twice with either (i) glycolysis stress test media [Seahorse Base Media w/o phenol red (Agilent Technologies) supplemented with 2mM L-glutamine]; (ii) mito stress test media [Seahorse Base Media w/o phenol red supplemented with 10mM D-Glucose, 2mM L-glutamine and 2mM Na-Pyruvate (life technologies)]; or (iii) XF-mito fuel stress test media [Seahorse Base Media w/o phenol red supplemented 10mM D-Glucose, 2mM L-glutamine and 1m Na-Pyruvate] before incubation with assay medium for 1 h at 37°C in a non-CO_2_ incubator. Plates were then treated and assessed using the XF Glycolysis Stress Test Kit (Agilent, SEA103020100), the XF Cell Mito Stress Test Kit (Agilent, SEA103015100) or the XFp Mito Fuel Flex Test Kit (Agilent, SEA103260100), according to the manufacturers’ instructions. Briefly, primary murine macrophages were analysed in response to subsequent injections of 10mM glucose, 2µM oligomycin and 2-deoxy-glucose (2-DG) for the glycolysis stress test (ECAR); and 2µM oligomycin, 2µM fluoro-carbonyl cyanide phenylhydrazone (FCCP), and 0.5µM Rotenone/Antimycin A (Rote/AA) for the mitochondrial stress test (OCR). For the assessment of fatty acid oxidation dependency, the OCR of macrophages were analysed in response to subsequent injection of a fatty acid inhibitor (4µM Etomoxir, long chain fatty acid inhibitor), and a combination of glutamine (3µM Bis-2-(5-phenylacetamido-1,3,4-thiadiazol-2-yl) ethyl sulfide, BPTES, glutamate inhibitor) and glucose (2µM 2-Cyano-3-(1-phenyl-1H-indol-3-yl)-2-propenoic acid, UK5099, pyruvate carrier blocker) inhibitors. The percentage of maximal capacity that is dependent upon FA oxidation was calculated from the maximal metabolic rate from each treatment. For the determination of fatty acid oxidation capacity, the OCR was measured from cells responding to a combination of glucose (2µM UK5099) and glutamine (3µM BPTES) inhibitors, followed by a long fatty acid inhibitor (4µM of Etomoxir). The fuel oxidation capacity was determined by the dependency to oxidize FA, and flexibility to use glucose (GLC) or glutamine (GLN).

### Normalization of cell number by CyQuant cell proliferation assay

A CyQuant cell proliferation assay kit (Invitrogen) was used to quantify cell numbers at the endpoint of live metabolic assays, following the manufacturer’s instructions. XFe-24 microplates containing cells were frozen at -80^0^C for 24 h upon completion of the metabolic flux assays. Prior to the quantification of cell numbers, the plates were allowed to reach room temperature before 200µL of CyQuant/cell-lysis buffer solution was added to each well. Fluorescence intensity was then measured using the Infinity M200 pro plate reader at ~480 nm excitation and ~520 nm emission maxima.

### Measurement of hexokinase activity

The hexokinase activity assay (Abcam-ab136957) was performed according to the manufacturer’s instructions. BMDMs were treated with FhHDM-1 (2.5µM or 15µM) for 1h, supernatants were discarded, and cells were washed twice with in RPMI 1640 medium (Gibco) containing 10% v/v heat-inactivated FCS (Gibco). Following this, cells were stimulated with LPS (20ng/mL) for 18h and then collected and homogenized in 100µL of ice-cold assay buffer. To allow for the homogenization process to occur, cells were incubated for 10 min on ice before centrifugation (10,000xg for 5 min). The supernatant was collected and assayed using the reaction mix, according to the instructions provided. The development of colour was then measured at 450 nm every 5 min for 30 min.

### Acyl-CoA mass spectrometry

To assess TCA cycle fuel utilization, BMDMs were treated with LPS (20ng/mL), IL-4 (20ng/mL), or FhHDM-1 (2.5µM) for 24h at 37^0^C and 5% CO_2_ followed by 1h of treatment with nutrient labelled/non-labelled media (100µM ^13^C_16_-potassium palmitate (Cambridge Isotope Laboratories) + 5mM glucose (Chem-supply), 100µM potassium palmitate (Sigma-Aldrich) + 5mM ^13^C_6_-glucose (Cambridge Isotope Laboratories), 100µM potassium palmitate + 5mM glucose in DMEM no glucose, no glutamine, no phenol red). Acyl-CoA analysis was performed by liquid chromatography- high resolution mass spectrometry (LC-HRMS) as previously described (43). Briefly, samples and calibration standards containing commercially available acetyl-CoA (Sigma-Aldrich) in 10% (w/v) trichloroacetic acid in water were spiked with ^13^C_3_^15^N_1_-acyl-CoA internal standards, sonicated for 12 × 0.5 s pulses, protein was pelleted by centrifugation at 17,000 ×g from 10 min at 4°C, then supernatant was extracted by solid-phase extraction using Oasis HLB 1cc (30 mg) SPE columns (Waters). Columns were washed with 1 mL methanol, equilibrated with 1 mL water, loaded with supernatant, desalted with 1 mL water, and eluted with 1 mL methanol containing 25 mM ammonium acetate. The purified extracts were evaporated to dryness under nitrogen then resuspended in 55 μL 5% (w/v) 5-sulfosalicylic acid in water. 5 µL of samples in 5% SSA were analyzed by injection of an Ultimate 3000 Quaternary UHPLC coupled to a Q Exactive Plus (Thermo Scientific) mass spectrometer in positive ESI mode using the settings described previously (44). Quantification of acyl-CoAs was *via* their predominant [(M-507) + H]^+^ product ions. Data were integrated using Tracefinder v4.1 (Thermo Scientific) software. Isotopic enrichment in tracing experiments was calculated by normalization to unlabelled control samples using the FluxFix calculator (45).

### Measurement of intracellular metabolites

BMDMs were cultured overnight with either LPS (20ng/mL), IL-4 (20ng/mL) or FhHDM-1 (2.5µM). After removal of the supernatants, cells were washed 3x with cold PBS. Cells were then harvested and snap frozen. Pellets were extracted by 80/20 Methanol/Water polar metabolite extraction. LC-HRMS was performed as previously described with minor modifications (46). Briefly, an Ultimate 3000 UHPLC equipped with a refrigerated autosampler (at 6°C) and a column heater (at 55°C) with a HSS C18 column (2.1 × 100 mm i.d., 3.5 μm; Waters, Milford, MA) was used for separations. Solvent A was 5 mM N,N-diisopropylethylamine (DIPEA) and 200 mM hexafluoro-2-propanol (HFIP) and solvent B was methanol with 5 mM DIPEA and 200 mM HFIP. The gradient was as follows: 100% A for 3 min at 0.18 mL/min, 100% A at 6 min with 0.2 mL/min, 98% A at 8 min with 0.2 mL/min, 86% A at 12 min with 0.2 mL/min, 40% A at 16 min and 1% A at 17.9 min-18.5 min with 0.3 mL/min then increased to 0.4 mL/min until 20 min. Flow was ramped down to 0.18 mL/min back to 100% A over a 5 min re-equilibration. For MS analysis, the UHPLC was coupled to a Q Exactive HF mass spectrometer (Thermo Scientific, San Jose, CA, USA) equipped with a HESI II source operating in negative mode. The operating conditions were as follows: spray voltage 4000 V; vaporizer temperature 200°C; capillary temperature 350°C; S-lens 60; in-source CID 1.0 eV, resolution 60,000. The sheath gas (nitrogen) and auxiliary gas (nitrogen) pressures were 45 and 10 (arbitrary units), respectively. Single ion monitoring (SIM) windows were acquired around the [M-H]^-^ of each analyte with a 20 *m/z* isolation window, 4 *m/z* isolation window offset, 1e^6^ ACG target and 80 ms IT, alternating with a Full MS scan from 70-950 *m/z* with 1e6 ACG, and 100 ms IT.

### Statistical analysis

In all cases, data are presented as means ± standard error of the means (SEMs) of a number of biological replicates (n). The comparison of data was determined by either an unpaired, two tailed Welch’s t-test or a one-way ANOVA with Tukey’s multiple comparison test using GraphPad Prism 7 software (GraphPad). Statistical significance was considered as a p-value<0.05.

## Results

### FhHDM-1 directs macrophages to utilise oxidative phosphorylation, fuelled by fatty acids

**Figure 1:**
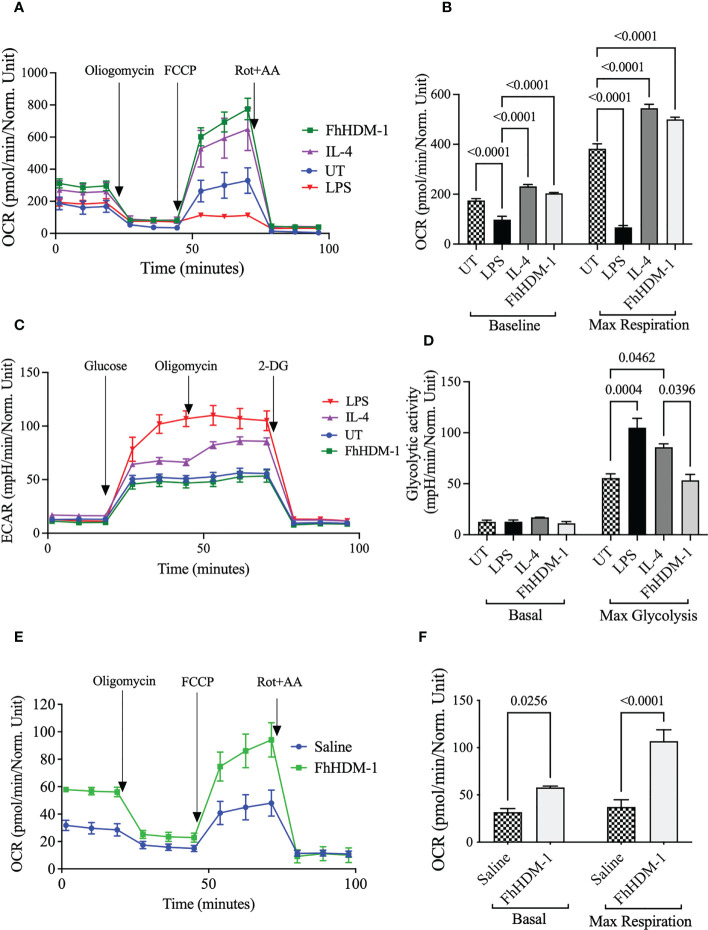
Unlike IL-4, treatment of macrophages with FhHDM-1 enhances oxidative phosphorylation without modulating glycolytic activity. **(A)** BMDMs were either untreated (UT) or treated with LPS (20ng/ml), IL-4 (20ng/ml), or FhHDM-1 (2.5µM) for 24h (n=5). The oxygen consumption rate (OCR) was measured at basal levels and following sequential treatments with oligomycin, FCCP, and rotenone/antimycin A (Rot+AA) to determine **(B)** the maximum respiratory capacity of cells. **(C, D)** BMDMs were cultured with media (UT), FhHDM-1 (2.5µM), LPS (20ng/mL) or IL-4 (20ng/mL) for 24h (n=5). Then glycolytic activity was determined by measuring **(C)** the extracellular acidification rate (ECAR) and **(D)** maximum glycolytic activity as cells were treated with glucose, oligomycin and 2-DG (2-deoxy-glucose). Statistical significance was determined by a one-way ANOVA with Tukey’s multiple comparison test. Data is representative of five independent experiments and is presented as means ± SEMs. **(E)** OCR was measured at basal levels and following sequential treatments with oligomycin, FCCP, and rotenone/antimycin A (Rot+AA) in peritoneal macrophages harvested from mice (n=10) that had received a single i.p. injection of either FhHDM-1 or saline. This data was used to **(F)** determine the maximum respiratory capacity of cells. Data is presented as means ± SEMs. Statistical significance was determined by an unpaired, two tailed Welch’s t-test.

To initially assess the effect of FhHDM-1, the metabolic activity of macrophages was determined in comparison to stimulation with LPS and IL-4. Bone marrow derived macrophages treated *in vitro* with FhHDM-1 displayed increased mitochondrial oxygen consumption rates (OCR), indicating an enhanced use of oxidative metabolism (**Figure 1A**) as compared to untreated cells, or cells treated with LPS. Furthermore, the increase in maximum respiratory capacity (MRC) observed in FhHDM-1 treated macrophages was equivalent to that seen in IL-4 induced M2 macrophages (**Figure 1B**). In contrast, examination of the extracellular acidification rate (ECAR) as a measure of glycolysis, suggested that unlike IL-4, FhHDM-1 had no effect on the glycolytic activity (**Figure 1C**), with FhHDM-1 treated macrophages showing the same maximum glycolytic activity as untreated cells, which was significantly less than values observed in cells treated with either IL-4 or LPS (**Figure 1D**). Validating this finding and verifying that the alteration to macrophage metabolism by FhHDM-1 occurs *in vivo*, peritoneal macrophages isolated from mice 1h after an intraperitoneal injection of FhHDM-1 also showed a similarly significant increase in OCR as compared to macrophages from mice that received saline (**Figure 1E, F**).

**Figure 2:**
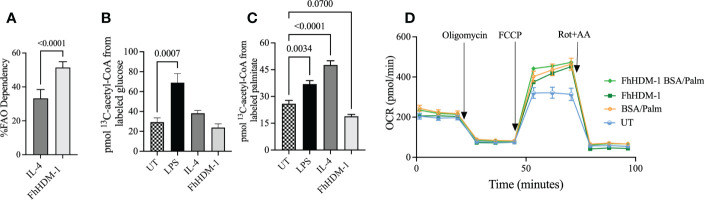
Oxidative phosphorylation induced by FhHDM-1 has a greater dependency on fatty acids than IL-4 driven metabolic activity. **(A)** The dependency of cells on fatty acids as fuel for mitochondrial respiration was determined by measuring the OCR in cells treated with either IL-4 (20ng/ml) or FhHDM-1 (2.5µM) in the presence of inhibitors of fatty acid oxidation or glycolysis and glutamine. **(B, C)** The incorporation of ^13^C into acetyl-CoA was determined by mass spectrometry analysis of BMDMs that had been untreated (UT) or cultured with FhHDM-1 (2.5µM), IL-4 (20ng/mL), or LPS (20ng/mL) for 24h followed by 1h in the presence of ^13^C_16_-potassium palmitate of ^13^C_16_-glucose (n=6). **(D)** The ability of cells to utilise exogenous fatty acids was determined by measuring the OCR levels in BMDMs cultured with 2.5µM FhHDM-1 in the presence of BSA/Palmitate (n=4). Statistical significance was determined by either an unpaired, two tailed Welch’s t-test or a one-way ANOVA with Tukey’s multiple comparison test. Data is representative of two-five independent experiments and is presented as means ± SEMs.

To investigate the underlying changes that were driving this switch in metabolism, the fuel preferences and flexibilities of macrophages were assessed. In FhHDM-1 treated macrophages the OCR had a strong dependency for fatty acid oxidation (FAO) (**Supplementary Figure S1**). In fact, FhHDM-1-treated macrophages were significantly more dependent on FAO to drive OCR than IL-4 treated cells (**Figure 2A**). Tracing ^13^C incorporation into the central metabolite acetyl-CoA supported the observation that FhHDM-1 treated cells did not display increased glucose metabolism (**Figure 2B**). However, unexpectedly this analysis also indicated that FhHDM-1 treated macrophages did not utilise the carbon provided by exogenous fatty acids (**Figures 2C**). Furthermore, the addition of exogenous palmitate did not augment metabolic respiration induced by FhHDM-1 treatment (**Figure 2D**). Although seemingly contradictory to the measure of enhanced OCR in response to FhHDM-1, these results suggested that the metabolic activity of macrophages induced by FhHDM-1 may be reliant on the oxidation of endogenous fatty acids.

### The FhHDM-1 induced metabolic switch to oxidative phosphorylation in macrophages is associated with an increase in fatty acid synthesis concomitant with glutaminolysis

**Figure 3:**
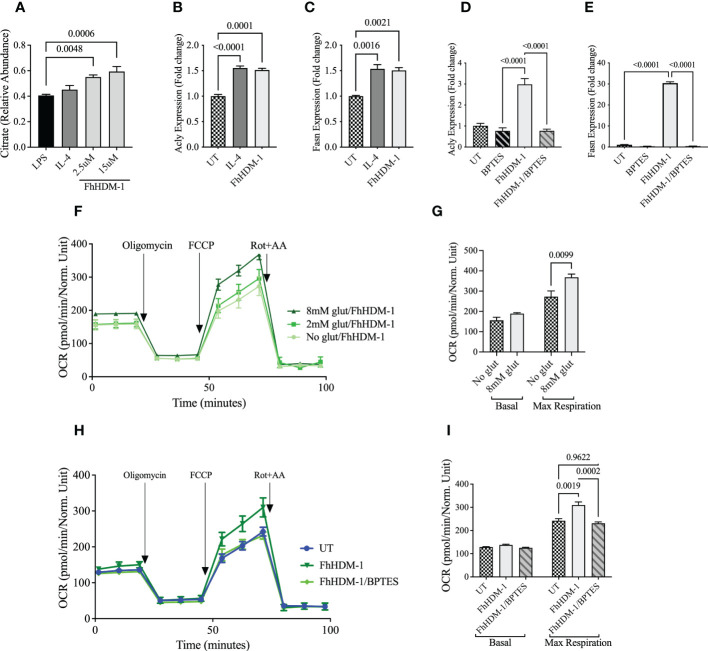
Oxidative phosphorylation induced by FhHDM-1 is fuelled by endogenous fatty acids synthesised *via* glutaminolysis. **(A)** BMDMs were treated with LPS (20ng/mL), IL-4 (20ng/mL) or FhHDM-1 (2.5µM and 15µM). The intracellular levels of citrate were quantified by mass spectrometry after metabolite extraction (n=6), and **(B–E)** The differential expression of Acly and Fasn was quantified by qRT-PCR in lysates of cells (n=3) that were untreated, or treated with IL-4, FhHDM-1(2.5µM), or a combination of FhHDM-1 and the glutamate inhibitor, BPTES (10µM), for 6h. **(F)** Glutamine dependency was determined by measuring the OCR in BMDMs treated with FhHDM-1 (2.5µM) for 6h in glutamine replete (2mM or 8mM) or depleted media for 18h (n=4). The basal oxygen consumption rate (OCR) was initially determined, and then measured following sequential treatments with oligomycin, FCCP, and rotenone/antimycin A (Rot+AA) to determine **(G)** the maximum respiratory capacity of cells. **(H, I)** The requirement for glutamine by FhHDM-1 treated cells was validated by measuring the **(H)** OCR and the **(I)** maximum respiratory capacity in BMDMs cultured in media (UT), or in the presence of FhHDM-1 (2.5µM) or a combination of FhHDM-1 and the glutamate inhibitor, BPTES (10µM), for 18h. Statistical significance was determined by a one-way ANOVA with Tukey’s multiple comparison test. Data is representative of three independent experiments and is presented as means ± SEMs.

The carbon required for the *de novo* synthesis of fatty acids can be derived from glutamine. Glutaminolysis leads to the production of α-ketoglutarate (α-KG), which is converted to citrate either *via* the TCA cycle or by reductive carboxylation. Cytoplasmic citrate is then converted to acetyl-CoA for fatty acid synthesis (44, 47). The observed increase in citrate abundance in macrophages treated with FhHDM-1, as compared to either LPS or IL-4 treatment (**Figure 3A**), coupled with the increase in expression levels of two key enzymes (fatty-acid synthase; FASN, ATP citrate lyase; ACLY) involved in fatty acid synthesis (**Figures 3B, C**) supports the likelihood that endogenous fatty acids were being synthesized for subsequent oxidation. The possibility that glutaminolysis was driving this pathway was confirmed by the observation that the increased expression of FASN and ACLY by FhHDM-1 was reversed in macrophages by the addition of the glutaminase inhibitor, bis-2-(5-phenylacetamido-1,3,4-thiadiazol-2-yl) ethyl sulphide (BPTES **Figures 3D, E**). The fact that FhHDM-1 was reprogramming macrophages to utilize glutamine as a major carbon source to fuel metabolic activity was further supported by the observation that the OCR induced by FhHDM-1 in macrophages was significantly enhanced under glutamine replete culturing conditions (**Figures 3F, G**), and inhibited by the addition of the BPTES (**Figures 3H, I**).

### FhHDM-1 induced glutaminolysis mediates a reduction in glycolytic flux and a regulation of pro-inflammatory responses

**Figure 4:**
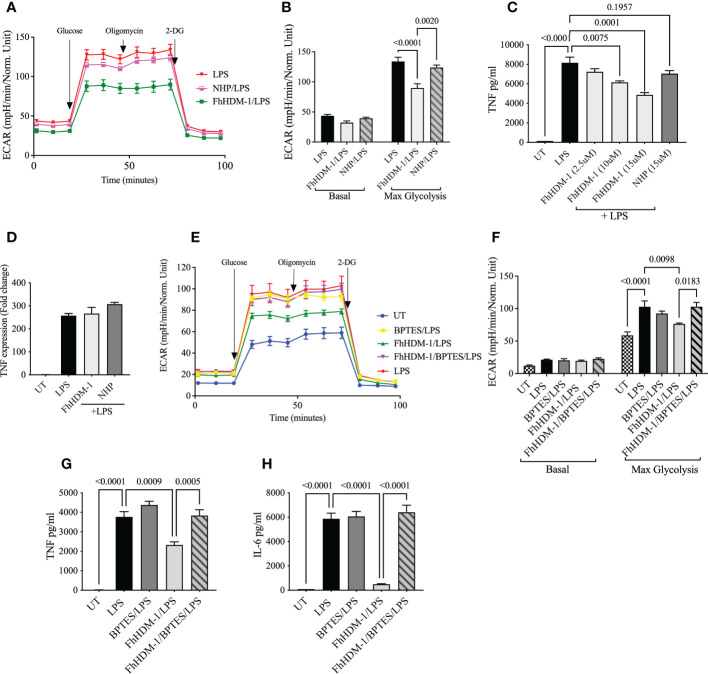
FhHDM-1 induced glutaminolysis mediates a reduced inflammatory response and decreased glycolysis, **(A)** BMDMs were treated 20ng/mL LPS or a combination of FhHDM-1 (2.5µM) or NHP (2.5µM), and LPS for 24h (n=4). Then glycolytic activity was determined by measuring the extracellular acidification rate (ECAR) and **(B)** maximum glycolytic activity as cells were treated with glucose, oligomycin and 2-deoxy-glucose (2-DG). **(C, D)** Macrophages derived from the bone marrow of C57BL/6 mice were either untreated (UT) or cultured for 1h with FhHDM-1 (2.5µM, 10µM and 15µM) or NHP (15µM), and then washed and stimulated with 20ng/ml LPS (n=3). **(C)** The levels of TNF secreted after an overnight incubation were quantified by ELISA, and **(D)** the levels of TNF gene expression at 6h after LPS treatment were determined by qRT-PCR. **(E)** BMDMs were either untreated (UT) or treated with LPS (20ng/mL), the glutamate inhibitor BPTES (10µM)/LPS, FhHDM-1 (2.5µM)/LPS or FhHDM-1/BPTES/LPS for 18h (n=4). The glycolytic activity was determined by measuring the extracellular acidification rate (ECAR) and **(F)** the maximum glycolytic capacity of cells after subsequent treatment with glucose, oligomycin and 2-deoxy-glucose (2-DG). **(G, H)** BMDMs were cultured overnight with LPS (20ng/mL), BPTES (10µM)/LPS, FhHDM-1 (15µM)/LPS, or FhHDM-1/BPTES/LPS. **(G)**The secreted TNF, **(H)** or IL-6 protein was quantified in the culture media by ELISA (n=3). Data is representative of two-five independent experiments and is presented as means ± SEMs. Statistical significance was determined by a one-way ANOVA with Tukey’s multiple comparison test.

When pro-inflammatory cytokines are not required due to the resolution of an inflammatory response or at the initiation of immune tolerance, the metabolic activity of cells is typically switched from glycolysis to predominantly OXPHOS. Thus, given the observed redirection of metabolic activity in FhHDM-1 treated cells, we hypothesised that FhHDM-1 may be hijacking this mechanism of immune-regulation to prevent pro-inflammatory immune responses in macrophages. In agreement with this, macrophages treated with FhHDM-1 showed significantly lower glycolytic activity in response to a subsequent stimulation with LPS (**Figures 4A, B**). Macrophages treated with FhHDM-1 also displayed significantly lower levels of hexokinase-1 activity and hypoxia-inducible factor 1α (HIF-1α) expression (**Supplementary Figure S2**) after stimulation with LPS. As both enzymes contribute to the initiation and regulation of the glycolytic pathway (48), this finding corroborated the observed reduction in glycolysis. This outcome was dependent on the ability of FhHDM-1 to inhibit vATPase, as an inactive derivative of the FhHDM-1 peptide [NHP-1 (36);] exerted no effect on the metabolic activity of macrophages (**Figures 4A, B**).

As expected, the amount of TNF protein secreted by macrophages in response to stimulation with bacterial LPS was significantly reduced by FhHDM-1, but not NHP-1, in a dose dependent manner (**Figure 4C**). However, the expression levels of *tnf* mRNA remained unchanged, even at the highest concentration of FhHDM-1 used (**Figure 4D**). Although apparently paradoxical, this result upholds the hypothesis that FhHDM-1 treatment of macrophages is reducing glycolysis, as this metabolic pathway mediates the post-transcriptional regulation of TNF. When glycolytic flux is reduced, glyceraldehyde-3-phosphate dehydrogenase (GAPDH), an enzyme in the glycolytic pathway, remains bound to TNF mRNA thereby repressing its translation (49) as observed in macrophages treated with FhHDM-1. To demonstrate that this switch in metabolic activity is not specifically associated with a reduction in TNF production, levels of other pro-inflammatory cytokines were also quantified. This analysis showed that the treatment of macrophages with FhHDM-1 also significantly reduced the secretion of IL-6, IL-1β, IL-12p70 and MCP-1 (**Supplementary Figure S3**).

It has been reported that the anti-inflammatory cytokine IL-10 has the capacity to regulate the expression of pro-inflammatory responses by inhibiting inflammatory induced glycolysis and promoting OXPHOS (26). However, FhHDM-1 treated macrophages showed no significant increase in the production of IL-10 in response to LPS stimulation compared to untreated cells (**Supplementary Figure S3**). This observation further supported the premise that FhHDM-1 was specifically targeting the pro-inflammatory response in macrophages by altering their metabolism, rather than exerting indirect effects through the increased production of regulatory cytokines, such as IL-10.

To mechanistically link this regulation of glycolysis and pro-inflammatory cytokines by FhHDM-1 to the metabolic conversion to glutaminolysis, both ECAR and cytokine secretion were measured in macrophages treated with FhHDM-1 and stimulated with bacterial LPS in the presence of the glutaminase inhibitor, BPTES. The presence of BPTES neither modulated the induction of glycolysis nor the production of TNF or IL-6 in response to LPS stimulation. However, the addition of BPTES to FhHDM-1 treated macrophages completely reversed both the inhibition of glycolysis and the suppression of LPS-induced TNF and IL-6 production, with levels of both restored to those observed in the LPS treated cells (**Figures 4E–H**). This evidence supports the conclusion that FhHDM-1 induced glutaminolysis is mediating the regulation of inflammatory responses in macrophages.

### The regulation of inflammation by FhHDM-1 is associated with an increased abundance of α-KG, is dependent on fatty acid oxidation, and is independent of M2 polarization

Based on current understanding, the induction of glutaminolysis in cells could be regulating the pro-inflammatory response in three ways. Firstly, the simple switch of metabolic activity from glycolysis to OXPHOS may be sufficient to prevent the synthesis of sufficient ATP and metabolites to support the pro-inflammatory response (7, 8). Secondly, although the specific mechanisms are unknown, an increase in FAO has been shown to reduce pro-inflammatory responses in macrophages and to enhance the differentiation to an M2 phenotype (23, 25, 50, 51). Finally, the accumulation of α-KG supports the transcriptional activation of M2 gene expression, an outcome which appears to be dependent on the ratio of α-KG:succinate. Independent to this, the accumulation of α-KG also impairs pro-inflammatory responses and promotes immune tolerance (52).

**Figure 5:**
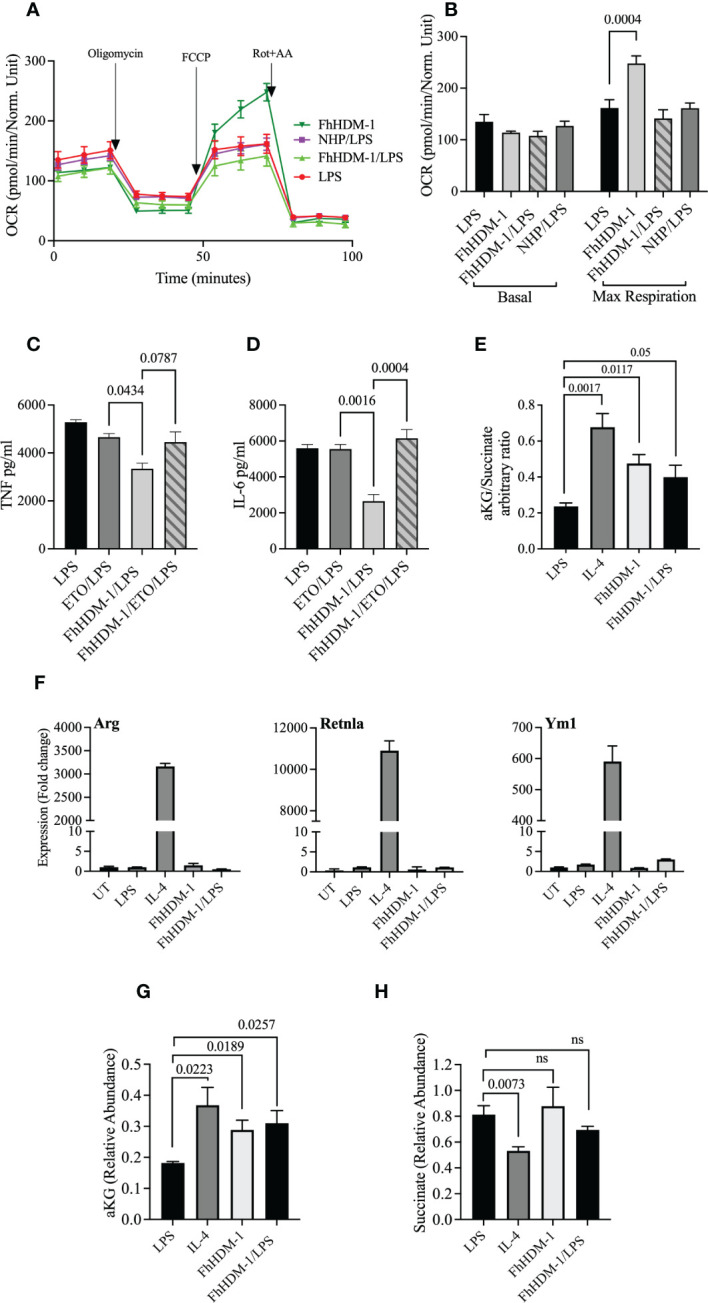
The regulation of inflammatory response by FhHDM-1 is associated with an increased αKetoglutarate : Succinate ratio, is dependent on FAS, and is independent of a switch to an M2 phenotype. **(A)** BMDMs were either untreated (UT) or treated with LPS (20ng/ml), FhHDM-1 (2.5µM), FhHDM/LPS or NHP (2.5µM)/LPS for 18h (n=5). The oxygen consumption rate (OCR) was measured at basal levels and following sequential treatments with oligomycin, FCCP, and rotenone/antimycin A (Rot+AA) to determine **(B)** the maximum respiratory capacity of cells. **(C)** BMDMs were treated with LPS (20ng/mL), LPS and Etomoxir (ETO) the inhibitor of fatty acid oxidation (5µM), FhHDM-1 (2.5µM)/LPS or FhHDM-1/ETO/LPS for 18h (n=3). The quantity of TNF, and **(D)** IL-6 released into the culture media was quantified by ELISA. **(E)** The intracellular ratio of α-ketoglutarate(α-KG):succinate in BMDMs treated overnight with LPS (20ng/mL), IL-4 (20ng/mL), FhHDM-1 (15µM), or a combination of FhHDM-1 and LPS (n=6) was calculated from the abundance of the individual metabolites as quantified by mass spectroscopy. **(F)** The differential expression of Arg1, Retnlα, and Ym1 was measured by qRT-PCR in cell lysates of BMDMs that were either untreated (UT) or cultured with LPS (20ng/mL), IL-4 (20ng/mL), FhHDM-1 (15µM), or a combination of FhHDM-1 and LPS for 6h (n=3). **(G)** Intracellular levels of α-KG and **(H)** succinate in BMDMs treated overnight with LPS (20ng/mL), IL-4 (20ng/mL), FhHDM-1 (15µM) or FhHDM-1 and LPS (n=6) was measured by mass spectrometry after metabolite extraction. Data is representative of three independent experiments and is presented as means ± SEMs. Statistical significance was determined by a one-way ANOVA with Tukey’s multiple comparison test. ns, not significant.

Although FhHDM-1 induced an increase in OXPHOS metabolism in resting/unstimulated macrophages (**Figure 1A**), a similar effect was not observed in FhHDM-1 treated macrophages that were also stimulated with LPS (**Figures 5A, B**). This outcome suggests that the inhibition of glycolysis, and the subsequent reduction in inflammatory cytokine production in response to LPS stimulation, was independent of OXPHOS metabolism induced by FhHDM-1. However, the inhibition of FAO by Etomoxir in FhHDM-1 treated macrophages restored the levels of both TNF and IL-6 produced in response to LPS stimulation (**Figures 5C, D**).

In addition, and supporting the evidence of increased glutaminolysis, treatment of macrophages with FhHDM-1 resulted in an increased ratio of α-KG:succinate (**Figure 5E**). However, in contrast to IL-4 stimulation, no significant increase in the expression of the M2 markers arginase 1 (Arg1), Retnlα or chitinase 3-like-3 was observed in macrophages treated either with FhHDM-1 alone, or in combination with LPS (**Figure 5F**). Unlike the treatment of macrophages with IL-4, in which the abundance of both α-KG and succinate are altered, the increased ratio of α-KG:succinate in FhHDM-1 treated macrophages is solely attributable to an increase in the levels of α-KG, with no significant change in the levels of succinate, in comparison to cells stimulated with LPS (**Figure 5G, H**). These observations demonstrate that the regulation of inflammatory responses induced by FhHDM-1 is mediated by an increase in α-KG *via* glutaminolysis, and an increase in FAO, independent of polarization to an M2 phenotype.

## Discussion

Emerging evidence indicates that mitochondrial metabolism has a defining role in regulating the balance of inflammatory versus tolerant macrophages (53, 54). In this study, we have shown that the parasite derived peptide, FhHDM-1, reprograms the metabolic activity of macrophages to regulate the production of TNF and IL-6 in response to simulation with an inflammatory ligand. Specifically, macrophages treated with FhHDM-1 were reliant upon OXPHOS fuelled by fatty acids and supported by elevated levels of α-KG produced by glutaminolysis. In contrast to IL-4 treated macrophages, the FhHDM-1 induced increase in FAO dependent OXPHOS metabolism was not associated with the induction of an M2 phenotype. However, the switch to glutamine metabolism prevented the induction of glycolysis and inhibited the activation of a pro-inflammatory M1 phenotype in response to subsequent stimulation with LPS.

We have previously shown that FhHDM-1 localizes to macrophage endolysosomes where it inhibits vATPase (36, 37). In the current study, treatment of macrophages with an inactive variant of FhHDM-1 (NHP) exerted no effect on the inflammatory response or the metabolic activity, thereby indicating that inhibition of lysosomal vATPase is required. This central role for the lysosome explains the difference in metabolic activity observed between FhHDM-1 and IL-4 treated macrophages, as the OXPHOS induced by IL-4 was reportedly dependent on lysosomal lipolysis of exogenous fatty acids (23). As the lysosomes of FhHDM-1 treated macrophages would be less acidic (due to inhibition of vATPase activity), lipolysis would be inhibited, and consequently the macrophages become more metabolically reliant on endogenous fatty acids synthesized from glutamine.

The premise that FhHDM-1 induced vATPase inhibition is modulating macrophage metabolism is corroborated by comparison to the biological activity of the vATPase inhibitor, bafilomycin A1 (BafA1). Like FhHDM-1, macrophages treated with BafA1 displayed a metabolic preference for OXPHOS, which was attributable to protons being rechannelled to the mitochondria due to impaired acidification of lysosomes (55). While the fuel source supporting this metabolic switch was not identified, Huh7 liver cells treated with the vATPase inhibitor, Archazolid-A, also displayed a metabolic shift to OXPHOS, which was shown to be glutamine dependent (56). Furthermore, increased cytosolic acidification in human fibroblasts was shown to induce the expression of glutaminase-1 and drive a switch towards glutaminolysis (57). It has been proposed that this alteration in metabolic activity is required in response to the change in cellular pH, as the ammonia produced by glutaminoysis neutralises the cytosolic pH to prevent cell death (57).

It has now been established that α-KG, another product of glutaminolysis, functions as a metabolic checkpoint for the reprogramming of macrophage phenotype and functional activity (52, 58, 59). Although the mechanism by which α-KG induces immune tolerance has not been fully elucidated, it has been shown that as a co-factor for the histone demethylase KDM5, it mediates the demethylation of active histone marks (H3K4me1/3) to reduce the expression of pro-inflammatory cytokines in macrophages (58). However, FhHDM-1 treated cells did not show any change in the expression of TNF, suggesting an alternative mechanism. An increased abundance of α-KG has also been shown to reduce the expression of DNMT3B, a DNA methyltransferase which mediates the differentiation of M1 macrophages (60, 61). In addition, the reduced expression of DNMT3B leads to a correlative reduction in the expression levels of dynamin-1-like protein (Drp1) (60). This is notable as Drp1 is an enzyme that catalyzes mitochondrial fission, a morphology that is associated with inflammation and enhanced glycolysis (55, 62). Consistent with this scenario, Drp1 is enriched on mitochondria in LPS-activated macrophages and is required for the induction of glycolysis, and the selective expression and/or secretion of pro-inflammatory cytokines. Like the outcome from FhHDM-1 treatment, silencing Drp1 in macrophages resulted in the translational, but not transcriptional, regulation of TNF expression (62, 63). This pathway proposes an intriguing link between glutaminolysis, mitochondrial dynamics, and the regulation of inflammatory responses.

The induction of glutaminolysis by FhHDM-1 also promoted an enhanced OXPHOS with a dependency on FAO. While the data presented here indicated that FAO was reliant on the synthesis of endogenous fatty acids, future inhibitory studies will be required to definitively connect FAS to FAO in FhHDM-1 treated cells. Nevertheless, a central functional role for FAO was established, as the inhibition of FAO in FhHDM-1 treated macrophages reduced the peptide’s regulatory effect, restoring the production of TNF and IL-6 in response to LPS stimulation. This outcome may suggest that the accumulation of α-KG is mediating the synthesis of endogenous fatty acids for subsequent oxidation, and it is in fact this pathway that is regulating the induction of glycolysis and production of pro-inflammatory cytokines, rather than a direct role for α-KG. However, a switch in mitochondrial dynamics from fission to fusion promoted by an increase in α-KG (60), is also required to support FAO and OXPHOS. Thus, additional studies will be required to elucidate the functional activity of the FhHDM-1 induced abundance of α-KG, and to tease apart the relative contributions of α-KG and FAO in mediating the FhHDM-1 regulation of the pro-inflammatory response of macrophages.

Nonetheless, the current literature combined with the data presented in this study suggest a mechanism by which the inhibition of lysosomal vATPase by FhHDM-1 causes a metabolic switch to glutaminolysis, resulting in the accumulation of α-KG and an increase in FAO. Consequently, the activation of an inflammatory response, mediated by glycolysis, is selectively regulated and the production of pro-inflammatory cytokines is inhibited. By impacting upon these innate regulatory mechanisms of mitochondrial metabolism, FhHDM-1 utilises a novel strategy for the regulation of inflammatory responses.

It is becoming widely acknowledged that metabolic reprogramming offers a unique strategy to fine-tune the functional activities of macrophages to limit inflammation, as opposed to globally suppressing inflammatory responses (1). This approach would allow for the targeted inhibition of pro-inflammatory pathologies, without compromising the ability to mount immune responses necessary for infection clearance and immunity after vaccination. Such a strategy represents a significant advantage over current immune therapeutic regimes, which are less targeted and accordingly induce undesirable global immune suppression. The demonstration that FhHDM-1 preferentially targets macrophages *in vivo* to metabolically reprogram them towards immune tolerance rather than an anti-inflammatory phenotype, makes this peptide an attractive therapeutic candidate for the multitude of conditions mediated by dysregulated or chronic inflammation.

## Data availability statement

The original contributions presented in the study are included in the article/**Supplementary Material**. Further inquiries can be directed to the corresponding author.

## Ethics statement

The animal study was reviewed and approved by University of Technology Sydney Animal Care and Ethics Committee.

## Author contributions

Conceptualization, SQ and SD. Methodology, SQ, NS, BO and SD. Formal Analysis, SQ, AT, EK and NS. Investigation, SQ, NS and SD. Writing – Original Draft, SQ and SD. Writing – Review and Editing, SQ, NS, BO and SD. Funding Acquisition, SD. All authors contributed to the article and approved the submitted version.
