# Supplementary material for: The helminth derived peptide FhHDM-1 redirects macrophage metabolism towards glutaminolysis to regulate the pro-inflammatory response
Source: Front Immunol. 2023 Jan 25;14:1018076. doi: 10.3389/fimmu.2023.1018076 (PMC9905698; doi:10.3389/fimmu.2023.1018076)
Supplement: Supplementary file 1 [file DataSheet_1.docx]

**Supplementary Tables and Figures:**

**Supplementary Table S1:**

| Oligonucleotides | | |
| --- | --- | --- |
| Mouse TNF | Applied Biosystems | Cat: Mm00443258_m1 |
| Mouse Arg1 primer | Applied Biosystems | Cat: Mm00475988-m1 |
| Mouse Retnla primer | Applied Biosystems | Cat: Mm0045109-m1 |
| Mouse Ym1 primer | Applied Biosystems | Cat: Mm04213363-u1 |
| Mouse HIF1α primer | Applied Biosystems | Cat: Mm00468869_m1 |
| Mouse Acly primer | Applied Biosystems | Cat: Mm01302282_m1 |
| Mouse Fasn primer | Applied Biosystems | Cat: Mm00662319_m1 |

**Supplementary Figure S1**

BMDMs were treated with IL-4 (20ng/mL) or FhHDM-1 (2.5µM) for 24h. (A) The oxygen consumption rate (OCR) was measured after treatment with combination of BPTES and UK5099 (a) followed by treatment with Etomoxir (b) to determine the metabolic capacity, or with Etomoxir (c) followed by BPTES and UK5099 (d) for fatty acids (FA) dependency. (B) The percentage of flexibility to use glucose and glutamine was calculated by the fuel capacity minus the dependency to oxidise FA. Data is representative of three independent experiments and is presented as means + SEMs. Statistical significance was determined by an unpaired parametric student *t*-test (two tailed, Welch’s correction). Data is representative of three independent experiments and is presented as means + SEMs.

**Supplementary Figure S2**

(A) Hexokinase activity was measured in BMDMs that had been untreated (UT) or stimulated overnight with 20ng/mL LPS only, or LPS in combination with FhHDM-1 (2.5µM and 15µM) (n=5). (B) RT-qPCR was used to quantify the expression levels of HIF1α in BMDMs that were untreated (UT) or cultured with 20ng/ml LPS, or LPS in combination with FhHDM-1 (2.5µM and 15µM) (n=3). Data is representative of three independent experiments and is presented as means + SEMs. Statistical significance was determined by an unpaired parametric student *t*-test (two tailed, Welch’s correction).

**Supplementary Figure S3**

(A) BMDMs were either untreated (UT) or treated FhHDM-1 (15µM) and then stimulated with LPS (20ng/ml) for 18h (n=4). The levels of cytokines secreted into the culture media were quantified by ELISA. Data is representative of two independent experiments and is presented as means + SEMs. Statistical significance was determined by an unpaired parametric student *t*-test (two tailed, Welch’s correction) to compare the effect of FhHDM-1 to untreated cells.
